# Region-Specific Apoptosis-Related Gene Expression Is Uncoupled from Viral RNA Load in Canine Distemper Neuropathogenesis

**DOI:** 10.3390/v18070720

**Published:** 2026-06-30

**Authors:** Bruno Benetti Junta Torres, Bernardo De Caro Martins, Luana de Sousa Ribeiro, Alessandra Silva Dias Campos, Marcos Bryan Heinemann, Graciela Kunrath Lima, Eliane Gonçalves de Melo

**Affiliations:** 1 Escola de Veterinária e Zootecnia, Universidade Federal de Goiás, Goiânia 74691-835, Goiás, Brazil; 2 Escola de Veterinária, Universidade Federal de Minas Gerais, Belo Horizonte 31270-901, Minas Gerais, Brazil; bernardodcmartins@hotmail.com (B.D.C.M.); alessandradias@ufg.br (A.S.D.C.); grakl@ufmg.br (G.K.L.); eliane@vet.ufmg.br (E.G.d.M.); 3 Instituto de Ciência Agrárias, Universidade Federal dos Vales do Jequitinhonha e Mucuri, Unaí 38610-000, Minas Gerais, Brazil; luana.ribeiro@ufvjm.edu.br; 4 Faculdade de Medicina Veterinária e Zootecnia, Universidade de São Paulo, São Paulo 05508-270, São Paulo, Brazil; marcosbryan@usp.br

**Keywords:** dogs, canine distemper virus, morbillivirus, apoptosis, neuropathogenesis, viral RNA load, brain regions

## Abstract

Canine distemper virus (CDV) is a highly contagious morbillivirus associated with severe neurological disease in dogs. Although apoptosis is recognized as an important mechanism in CDV-associated neurodegeneration, the relationship between regional viral RNA load and apoptosis-related transcriptional responses remains poorly defined. This study aimed to quantify CDV RNA load and the expression of apoptosis-related genes, including the pro-apoptotic markers Bax, caspase-3 and caspase-8 and the anti-apoptotic marker Bcl-2, in the frontal cortex, hippocampus, and cerebellum of 21 dogs naturally infected with CDV, compared with four neurologically normal controls. Viral RNA load and gene expression were assessed by quantitative reverse transcription PCR (qRT-PCR). CDV RNA was detected in all brain regions of infected dogs, with no significant differences in viral RNA load among the frontal cortex, hippocampus and cerebellum (*p* > 0.05). In contrast, apoptosis-related gene expression showed a region-specific pattern. In the hippocampus, Bax (2.56-fold, *p* < 0.05), caspase-8 (4.33-fold, *p* < 0.05) and caspase-3 (2.01-fold, *p* < 0.05) were significantly upregulated in CDV-infected dogs compared with controls. In the cerebellum, Bax (2.07-fold, *p* < 0.05) and caspase-3 (1.97-fold, *p* < 0.05) were also increased, whereas no significant differences were observed in any apoptotic marker in the frontal cortex. Pearson correlation analysis revealed no significant association between regional viral RNA load and expression of Bcl-2, Bax, caspase-3 or caspase-8 in any of the brain regions examined. These findings suggest that CDV-associated apoptosis-related transcriptional responses in the central nervous system are region-dependent and are not linearly associated with local viral RNA load. This study provides new insights into the heterogeneous neuropathogenesis of CDV and supports the importance of regional brain susceptibility in viral encephalitis.

## 1. Introduction

Canine distemper virus (CDV) is a highly contagious and globally distributed Morbillivirus that affects domestic dogs and a wide range of wild carnivore species [1]. The neurological form of canine distemper is associated with substantial morbidity and mortality and is among the most severe clinical manifestations of the disease [2].

CDV neuropathogenesis is complex, and although numerous studies have extensively characterized the histopathological and clinical features of CDV-induced neurological disease, the relationship between lesion distribution and clinical signs remains inconsistent [3,4,5]. This lack of correlation complicates a comprehensive understanding of disease progression within the central nervous system (CNS).

Apoptosis, or programmed cell death, plays a central role in CDV-induced neurological damage and contributes significantly to neuronal loss and demyelination [6]. This tightly regulated process is primarily mediated by two major pathways: the intrinsic (mitochondrial) pathway, regulated by proteins such as Bax and Bcl-2, and the extrinsic pathway, triggered by death receptors and involving caspase-8 activation [7]. Studies have shown that CDV infection induces widespread apoptosis throughout the brain [8], with activation of both apoptotic pathways occurring regardless of clinical presentation [9].

Despite these advances, it remains unclear whether regional CDV RNA load is associated with apoptosis-related gene expression in distinct brain regions. This gap in knowledge limits a deeper understanding of CDV neuropathogenesis and hinders the identification of potential molecular targets for therapeutic intervention, particularly given the lack of effective treatments for canine distemper [7]. Therefore, the present study aimed to evaluate CDV RNA load and the expression of apoptosis-related genes (Bcl-2, Bax, caspase-3 and caspase-8) in the frontal cortex, hippocampus and cerebellum of naturally infected dogs. We hypothesized that regional viral RNA load would be positively associated with apoptosis-related gene expression and that this association would differ among brain regions.

## 2. Materials and Methods

### 2.1. Animals and Sample Collection

This study was conducted in accordance with the ethical guidelines for animal research (protocol number UFMG 32/2013). Brain tissue samples were collected from dogs that died or were euthanized because of advanced neurological canine distemper, confirmed by compatible clinical signs and molecular diagnosis. Four neurologically normal dogs that died from causes unrelated to CDV served as controls. These animals tested negative for CDV by both immunochromatographic assay and qRT-PCR. Samples were collected from three specific regions: the frontal cortex, hippocampus, and cerebellum. Tissues were immediately snap-frozen in liquid nitrogen and stored at −80 °C until processing.

### 2.2. RNA Extraction and Reverse Transcription

Total RNA was extracted from approximately 100 mg of brain tissue using TRIzol reagent (Invitrogen, Carlsbad, CA, USA), following the manufacturer’s instructions. Genomic DNA contamination was removed using the Turbo DNA-free™ Kit (Invitrogen, Carlsbad, CA, USA), and RNA purity and concentration were verified by spectrophotometry. One microgram of total RNA was reverse transcribed to complementary DNA (cDNA). For the analysis of apoptosis-related gene expression, cDNA synthesis was performed using SuperScript™ III First-Strand Synthesis SuperMix (Invitrogen, Carlsbad, CA, USA). For viral RNA load quantification, cDNA was synthesized using TaqMan™ Reverse Transcription Reagents (Invitrogen, Carlsbad, CA, USA). In both protocols, the reverse transcription conditions consisted of 25 °C for 10 min, 50 °C for 30 min, and 85 °C for 5 min.

### 2.3. qRT-PCR Analysis of Apoptosis-Related Gene Expression (Bax, Bcl-2, caspase-3, caspase-8)

Quantification of gene expression for Bcl-2, Bax, caspase-3, and caspase-8 was performed using a 7500 Real-Time PCR System (Applied Biosystems,. Waltham, MA, USA) with Platinum™ SYBR™ Green qPCR SuperMix-UDG (Invitrogen, Carlsbad, CA, USA). Reactions were performed in duplicate in a final volume of 20 μL. Thermal cycling conditions included an initial step at 50 °C for 2 min and 95 °C for 10 min, followed by 40 cycles of 95 °C for 15 s and 60 °C for 1 min. Dissociation curves were generated at the end of each run to confirm specificity of amplification. Primers were based on previous publications (Del Puerto et al. [9]) and are detailed in Table 1. The gene encoding ribosomal protein S26 (S26CF) was used as the endogenous control. Relative gene expression was calculated using the 2^−ΔΔCt^ method [10]. No-template controls (NTCs) and no-reverse-transcription controls (NRTs) were included in each run (negative controls), and synthetic DNA standards for each gene were used as positive controls.

### 2.4. qRT-PCR Analysis of CDV RNA Load

For CDV RNA quantification, qRT-PCR was performed using the 7300 Real-Time PCR System (Applied Biosystems. Waltham, MA, USA) and GoTaq^®^ qPCR Master Mix (Promega, Madison, WI, USA). Primers targeting the nucleocapsid (N) gene were used: CDV-For (5′-AGC TAG TTT CAT CTT AAC TAT CAA ATT-3′) and CDV-Rev (5′-TTA ACT CTC CAG AAA ACT CAT GC-3′), as described by Elia et al. [11]. A 287 bp fragment of the N gene was cloned into the pGEM^®^-T Easy vector (Promega, Madison, WI, USA) and transcribed in vitro using T7 RNA polymerase. The resulting RNA transcripts were purified, quantified by spectrophotometry, and serially diluted (10^3^ to 10^9^ copies/μL) to generate the standard curve. Each sample and standard were analyzed in duplicate with a final reaction volume of 20 μL. The cycling conditions consisted of 95 °C for 5 min, followed by 40 cycles of 95 °C for 15 s and 60 °C for 1 min. Dissociation curve analysis was performed at the end of the run. The qPCR demonstrated high linearity (R^2^ = 0.99), efficiency of 98.2%, and a slope of −3.366.

### 2.5. Statistical Analysis

Data analysis was performed using GraphPad Prism 5.0 (GraphPad Software, San Diego, CA, USA). Regional comparisons among the frontal cortex, hippocampus, and cerebellum within infected dogs were analyzed by one-way ANOVA followed by Tukey’s post hoc test. Comparisons between infected and control animals for each brain region were performed using unpaired t-tests. Normality and homogeneity of variances were assessed before parametric testing. Pearson correlation was used to evaluate linear associations between viral RNA load and gene expression values. Statistical significance was defined as *p* < 0.05.

Data analysis was performed using GraphPad Prism 5.0 (GraphPad Software, San Diego, CA, USA). Data are presented as mean ± standard error of the mean (SEM). Regional comparisons among the frontal cortex, hippocampus, and cerebellum within infected dogs were analyzed by one-way ANOVA followed by Tukey’s post hoc test. Comparisons between infected and control animals for each brain region were performed using unpaired t-tests. Normality and homogeneity of variances were assessed before parametric testing. Pearson correlation was used to evaluate linear associations between viral RNA load and gene expression values. Statistical significance was defined as *p* < 0.05.

## 3. Results

From a total of 90 dogs admitted with clinical suspicion of canine distemper during the study period, 21 cases met all inclusion criteria and were selected for analysis, together with four neurologically normal dogs that died from causes unrelated to CDV and served as controls.

### 3.1. Neurological Clinical Signs

The neurological signs observed in the 21 CDV-positive dogs are summarized in Table 2. The most frequent abnormalities were altered mentation (76%, 16/21) and motor deficits (76%, 16/21), including paresis and tetraparesis. Based on the neurological examination, neuroanatomical lesion localization was most attributed to the brainstem (47.6%, 10/21), followed by multifocal involvement (23.8%, 5/21), forebrain (14.3%, 3/21) and spinal cord (9.5%, 2/21). No dog showed a purely cerebellar syndrome. Several animals displayed combinations of vestibular ataxia, head tilt, positional strabismus, myoclonus, spinal hyperesthesia and seizures, reflecting the broad spectrum and multifocal nature of CDV-associated neurological disease.

### 3.2. Relative Genetic Expression Evaluation of Bcl-2, Bax, caspase-3, caspase-8 by qRT-PCR

**Figure 1:**
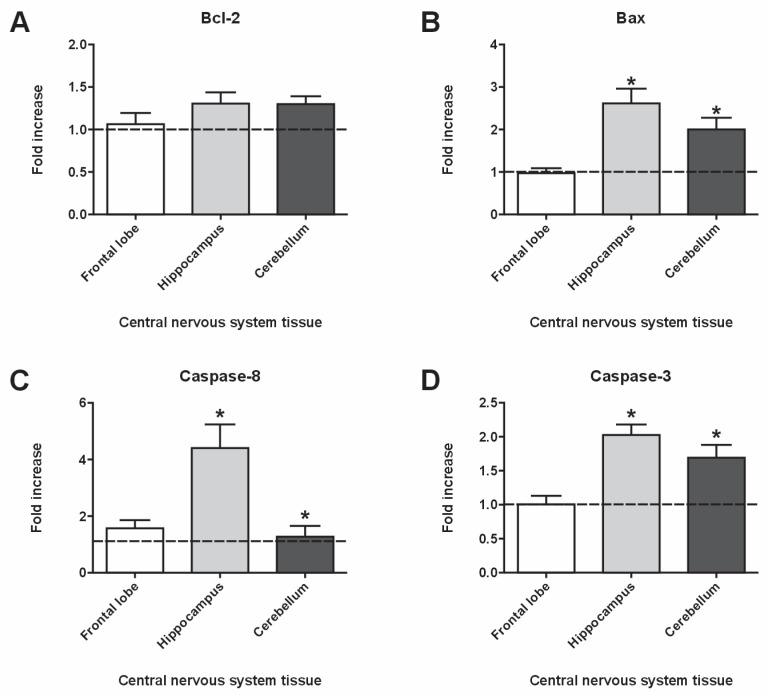
Relative expression of Bcl-2 (**A**), Bax (**B**), caspase-8 (**C**) and caspase-3 (**D**) in the frontal cortex, hippocampus and cerebellum of CDV-positive dogs and controls. Data are presented as mean fold-change, with the control group set as baseline (1.0). Error bars represent the standard error of the mean (SEM). * *p* < 0.05. Analysis was performed using GraphPad Prism 5.

Analysis of apoptosis-related gene expression revealed a region-specific pro-apoptotic transcriptional pattern in CDV-positive dogs (Figure 1). In the hippocampus, Bax (2.56-fold), caspase-8 (4.33-fold) and caspase-3 (2.01-fold) were significantly upregulated compared with controls (*p* < 0.05). In the cerebellum, Bax (2.07-fold) and caspase-3 (1.97-fold) were also significantly increased compared with controls (*p* < 0.05), whereas caspase-8 and Bcl-2 expression did not differ from the control group. In contrast, no statistically significant differences were detected in Bcl-2, Bax, caspase-3 or caspase-8 expression in the frontal cortex between CDV-positive and control dogs. No significant regional variation in gene expression was observed among the frontal cortex, hippocampus and cerebellum in the control group.

### 3.3. Viral RNA Load Quantification in the Brain by qRT-PCR

**Figure 2:**
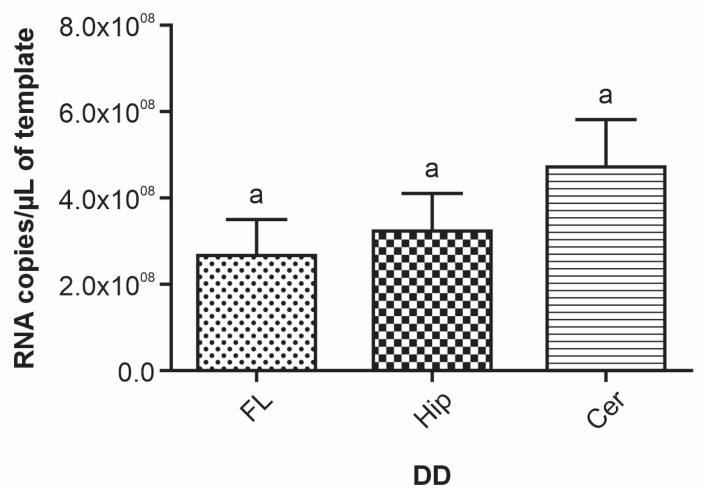
CDV RNA load in the frontal cortex (FL), hippocampus (Hip) and cerebellum (Cer) of CDV-positive dogs. Data are presented as mean values. Same lowercase letters indicate no differences among brain regions (*p* > 0.05). Error bars represent the standard error of the mean (SEM). Analysis was performed using GraphPad Prism 5.

Quantitative RT-PCR demonstrated high CDV RNA loads across all three brain regions analyzed in infected dogs. Viral RNA load in the frontal cortex ranged from 9.57 × 10^4^ to 1.19 × 10^9^ copies/µL in the hippocampus from 7.57 × 10^4^ to 1.11 × 10^9^ copies/µL, and in the cerebellum from 3.90 × 10^4^ to 1.32 × 10^9^ copies/µL. Although the cerebellum exhibited the highest mean viral RNA load (4.7 × 10^8^ copies/µL), followed by the hippocampus (3.2 × 10^8^ copies/µL) and frontal cortex (2.6 × 10^8^ copies/µL), no statistically significant differences were found among the three regions (*p* > 0.05; Figure 2).

### 3.4. Correlation Between Relative Genetic Expression of Apoptotic Factors and Viral RNA Load in the Brain

Pearson’s correlation analysis did not reveal any significant association between regional viral RNA load and the expression levels of Bcl-2, Bax, caspase-3 or caspase-8 in the frontal cortex, hippocampus or cerebellum of CDV-infected dogs (Table 3). Correlation coefficients (r) were low and non-significant in all comparisons, and the 95% confidence intervals consistently included zero, indicating absence of a linear relationship between viral burden and transcriptional activation of the apoptosis-related genes evaluated in the brain regions studied.

## 4. Discussion

The results of this study highlight the complex nature of CDV neuropathogenesis. We investigated CDV viral RNA load and apoptosis-related gene expression in selected brain regions of naturally infected dogs. Based on the known cytopathic potential of CDV [8,9,12], we initially hypothesized that regional viral RNA load would be positively associated with apoptosis-related gene expression. Consistent with previous reports, CDV RNA was detected in all evaluated brain areas [11]. However, contrary to our initial hypothesis, no significant correlation was observed between viral RNA load and the expression of Bcl-2, Bax, caspase-3 or caspase-8 in any of the regions examined. Furthermore, the apoptotic pathways triggered by CDV infection showed a distinct regional pattern: the cerebellum predominantly exhibited activation of the intrinsic pathway (Bax and caspase-3), whereas the hippocampus showed evidence of both intrinsic and extrinsic pathway involvement (Bax, caspase-3 and caspase-8).

Although CDV may display widespread distribution within the CNS, its quantitative distribution has been reported as highly heterogeneous, with high concentrations in the frontal and temporal cortices and the prorean gyrus [11]. In contrast, in our series no statistically significant differences in mean viral RNA loads were found among the frontal cortex, hippocampus and cerebellum. This apparent discrepancy may be explained, at least in part, by methodological differences, particularly the fact that Elia et al. based their observations on a single animal, whereas our analysis comprised 21 naturally infected dogs. In addition, the specific neuroanatomical regions selected for analysis differed between studies, and it is plausible that the extreme viral RNA load values documented by Elia et al. originated from cortical areas not encompassed within our sampling strategy.

To the best of our knowledge, this is the first study to use quantitative RT-PCR to evaluate regional viral RNA load in parallel with apoptosis-related gene expression in the brains of dogs naturally infected with CDV. The lack of correlation between neural apoptosis and viral RNA load observed here corroborates the findings of McQuaid et al. [13], who demonstrated no linear relationship between viral burden and the extent of apoptosis in central nervous system tissues from patients with measles virus-associated encephalitis. Given the close genetic relatedness between measles virus and CDV and their association with severe neurological disease in humans and animals, morbilliviruses are recognized as important pathogens causing severe disease in humans and animals [14]. Together, these data support the concept that Morbillivirus-induced neuronal death is largely driven by indirect mechanisms and host responses rather than by local viral replication intensity alone.

Apoptosis has an important role in neuronal cell death and demyelinating lesions [6], and its mechanisms involve complex extrinsic and intrinsic pathways [15], in which the balance between pro-apoptotic and anti-apoptotic proteins determines cell fate [7]. In the present study, analysis of apoptosis-related gene expression in the frontal cortex, hippocampus and cerebellum revealed region-specific responses. Notably, no statistically significant differences in apoptotic gene expression were observed in the frontal cortex. This contrasts with reports describing widespread apoptosis throughout the encephalon, including the cerebrum [8]. However, Pan et al. [8] did not specify the exact cortical regions analyzed and used histological and immunohistochemical approaches rather than regionalized quantitative gene expression. Differences in anatomical sampling, disease stage, viral strain and methodological approach may therefore contribute to the divergent observations.

In the cerebellum, we observed upregulation of Bax and caspase-3, indicating activation of the intrinsic apoptotic pathway, whereas caspase-8 did not differ significantly from controls. Conversely, the hippocampus exhibited increased expression of Bax, caspase-3 and caspase-8, suggesting the involvement of both intrinsic and extrinsic pathways, while Bcl-2 expression remained similar to that of the control group. These findings add to a growing body of evidence demonstrating the diverse and context-dependent nature of CDV-induced apoptosis. For example, in naturally infected dogs, Del Puerto et al. [9] reported increased expression of Bax, caspase-3, caspase-8 and caspase-9 in the cerebellum and concluded that apoptosis in this region occurs via both extrinsic (caspase-8) and intrinsic (caspase-9 and Bax) pathways. Our results partially overlap with those of Del Puerto et al. [9], reinforcing the central role of Bax and caspase-3 in cerebellar apoptosis, while suggesting that the extent and combination of pathway activation may vary among animals, possibly due to differences in disease stage, viral lineage, host background or sampling site within the cerebellum. A more comprehensive understanding of these apoptotic mechanisms may support future studies on antiviral and neuroprotective strategies for CDV infection.

Further illustrating this variability, in vitro studies of CDV infection have also reported distinct apoptotic signatures. In Vero cells infected with the CDV-Onderstepoort strain, Kajita et al. [12] showed activation of caspase-3 and caspase-8 without changes in Bcl-2 or Bax, indicative of a predominantly extrinsic pathway. In contrast, Singh and Deka [16], working with colorectal cancer cell lines, did not detect upregulation of caspases-3, -8 or -9 despite increased Bax and decreased Bcl-2, suggesting alternative or non-canonical apoptotic mechanisms. When considered together with the region-dependent transcriptional patterns observed in our study, these findings highlight the potential influence of distinct viral strains and host tissue specificities. CDV is known for its genetic diversity and varied pathogenic profiles across lineages [14]. Thus, it is likely that strain- and tissue-dependent variation contributes to the observed differences in apoptotic mechanisms triggered in distinct brain regions. A better understanding of how specific CDV lineages interact with neural cell populations and apoptotic cascades may be helpful for the development of new antiviral strategies and for exploring CDV-based virotherapy approaches.

The relationship between viral distribution, lesion localization and neurological signs in canine distemper remains incompletely understood. Although a direct link might be expected, post-mortem studies have demonstrated diffuse, multifocal lesions in dogs presenting with apparently focal clinical signs [4,5], and conversely, multifocal signs in animals with lesions confined to a single neuroanatomical site [3]. Histopathological studies have also highlighted a predominance of lesions in the cerebellum [17], even though overt cerebellar signs are relatively uncommon [4,18]. In the present study, CDV RNA was widely distributed throughout the CNS, and apoptosis-related gene expression was increased in the hippocampus and cerebellum but not in the frontal cortex. However, the neuroanatomical sampling in this study was limited to the frontal cortex, hippocampus, and cerebellum, and did not include the brainstem, the most frequent clinical neuroanatomic localization in this cohort (47.6% of cases). Consequently, the present data do not allow direct correlation between molecular findings and specific clinical syndromes, and this limitation should be considered when interpreting the region-specific patterns reported here. Future studies with broader regional sampling, particularly including the brainstem, and integrated molecular, histopathological and clinical analyses will be required to address this question.

This study has several limitations that should be considered. First, apoptosis was evaluated exclusively at the mRNA level by qRT-PCR. Although this technique is sensitive for detecting changes in apoptosis-related gene transcription [16], it does not confirm protein activation or cell death in situ. Nonetheless, the genes investigated here have previously been associated with apoptosis in CDV infection [8,9], and our primary objective was to explore their transcriptional relationship with regional viral RNA load. Second, the observational design and reliance on naturally occurring cases introduce potential selection bias toward more severe or advanced clinical manifestations, as well as heterogeneity in CDV strains and lesion patterns. Despite these constraints, this approach is valuable for characterizing disease progression under natural conditions, which cannot be fully reproduced in experimental models. Third, sample collection did not encompass all CNS regions relevant to the spectrum of clinical signs, including the brainstem and spinal cord, limiting the ability to link molecular changes to specific functional deficits. Finally, we did not characterize the viral strains circulating in our cases. Given that CDV virulence can vary according to the circulating lineage, strain-specific differences may influence viral load, lesion distribution and apoptotic responses among brain regions. Future work should correlate viral genotype, viral RNA load, gene and protein expression, and direct markers of apoptosis across multiple CNS regions, integrating these findings with detailed clinical and imaging data.

## 5. Conclusions

This study demonstrates a region-specific pro-apoptotic transcriptional response in the brains of naturally CDV-infected dogs, particularly in the hippocampus and cerebellum, where Bax, caspase-3 and caspase-8 (hippocampus) or Bax and caspase-3 (cerebellum) were significantly upregulated compared with neurologically normal controls. In contrast, no significant changes in apoptotic gene expression were detected in the frontal cortex, despite the presence of high viral RNA loads in all regions. No direct correlation was observed between regional viral RNA load and the expression of apoptosis-related genes in any of the brain regions examined. However, these findings are subject to important limitations. Apoptosis was assessed exclusively at the transcriptional level, CDV strains were not characterized, and the neuroanatomical sampling did not include the brainstem—the most frequent clinical localization in this cohort. Therefore, while our data indicate that regional apoptosis-related transcriptional responses occur independently of local viral RNA load in the frontal cortex, hippocampus, and cerebellum, this conclusion is restricted to the transcript level and to the specific brain regions and uncharacterized viral strains examined. Whether this independence holds at the protein level or extends to other neuroanatomical sites and defined CDV lineages remains to be determined. These findings support the concept that canine distemper neuropathogenesis involves region-dependent mechanisms and may be influenced by indirect factors such as neuroinflammation and the local microenvironment. Future studies combining viral genotyping, protein-level apoptosis markers, and expanded regional coverage, particularly including the brainstem, will be essential to validate and extend these observations.

## Figures and Tables

**Table 1:** Primers used for real-time PCR amplification of cDNA targeting apoptosis-related genes.

Gene	Primer (5′–3′ Sequence)
S26CF–Reference gene	forward: 5′-CGTGCTTCCCAACGTGTACGTGA-3′ reverse: 5′-CGATTCCGGACTACCTTGCTGTG-3′
BCL2	forward: 5′-CATGCCAAGAGGGAAACACCAGAA-3′ reverse: 5′-GTGCTTTGCATTCTTGGATGAGGG-3′
Caspase-8	forward: 5′-ACAAGGGCATCATCTATGGCTCTGA-3′ reverse: 5′-CCAGTGAAGTAAGAGGTCAGCTCAT-3′
Bax	forward: 5′-TTCCGAGTGGCAGCTGAGATGTTT-3′ reverse: 5′-TGCTGGCAAAGTAGAAGAGGGCAA-3′
Caspase-3	forward: 5′-TTCATTATTCAGGCCTGCCGAGG-3′ reverse: 5′-TTCTGACAGGCCATGTCATCCTCA-3′

**Table 2:** Neurologic clinical signs and lesion localization in 21 dogs naturally infected by canine distemper virus.

DOG #	AGE (MONTHS)	NEUROLOGIC DEFICITS RECORDED	NEUROANATOMIC LOCALIZATION
**1**	108	Altered mentation, head tilt, ambulatory tetraparesis, right positional strabismus	Brainstem
**2**	6	Altered mentation, ambulatory tetraparesis, seizures, myoclonus, right positional strabismus, reduced flexor withdrawal reflexes in all limbs	Multifocal: forebrain, brainstem, spinal cord
**3**	2	Altered mentation, head tilt, ambulatory hemiparesis, ventrolateral positional strabismus	Brainstem
**4**	36	Altered mentation, myoclonus, head tilt, ambulatory tetraparesis, right positional strabismus	Brainstem
**5**	12	Altered mentation, seizures	Forebrain
**6**	132	Altered mentation, vocalization, discrete ambulatory hemiparesis, discrete proprioceptive ataxia	Forebrain
**7**	2	Altered mentation, ambulatory hemiparesis, ventrolateral positional strabismus, spinal hyperesthesia	Multifocal: brainstem, spinal cord
**8**	24	Ambulatory tetraparesis, myoclonus, ventrolateral positional strabismus, spinal hyperesthesia	Multifocal: brainstem, spinal cord
**9**	24	Altered mentation, non-ambulatory tetraparesis, head tilt, vestibular ataxia, myoclonus, ventrolateral positional strabismus, spinal hyperesthesia	Multifocal: brainstem, spinal cord
**10**	6	Head tilt, vestibular ataxia, ambulatory tetraparesis, ventrolateral positional strabismus	Brainstem
**11**	12	Altered mentation, head tilt, ambulatory tetraparesis, ventrolateral positional strabismus	Brainstem
**12**	12	Altered mentation, head tilt, ambulatory tetraparesis, vestibular ataxia, ventrolateral positional strabismus	Brainstem
**13**	4	Seizures, myoclonus	Multifocal: forebrain, brainstem or spinal cord
**14**	144	Proprioceptive ataxia, spinal hyperesthesia	Spinal cord
**15**	48	Altered mentation, non-ambulatory tetraparesis, head tilt, vertical nystagmus, ventrolateral positional strabismus	Brainstem
**16**	60	Proprioceptive ataxia, ambulatory tetraparesis, myoclonus, spinal hyperesthesia	Spinal cord
**17**	60	Altered mentation	Forebrain or Brainstem
**18**	36	Altered mentation, vestibular ataxia, ambulatory tetraparesis, ventrolateral positional strabismus	Brainstem
**19**	12	Altered mentation, myoclonus, vestibular ataxia, ambulatory tetraparesis	Brainstem
**20**	24	Altered mentation, head tilt, vestibular ataxia, ambulatory tetraparesis	Brainstem
**21**	48	Altered mentation, ventrolateral positional strabismus	Brainstem

**Table 3:** Correlation between viral RNA load and apoptotic factors (Bcl-2, Bax, caspase-8, caspase-3) in the brain (frontal cortex, hippocampus, and cerebellum) of dogs naturally infected with canine distemper virus.

	APOPTOTIC FACTOR			
BRAIN REGION	Bcl-2	Bax	Caspase-8	Caspase-3
**Frontal cortex** ^a^	r = 0.469 ^a^ (−0.181 to 0.834)	r = 0.024 ^a^ (−0.614 to 0.644)	r = 0.538 ^a^ (−0.05 to 0.849)	r = 0.502 ^a^ (−0.186 to 0.860)
**Hippocampus** ^b^	r = −0.180 ^b^ (−0.704 to 0.470)	r = −0.297 ^b^ (−0.729 to 0.303)	r = −0.358 ^b^ (−0.825 to 0.401)	r = 0.301 ^b^ (−0.329 to 0.746)
**Cerebellum** ^c^	r = −0.072 ^c^ (−0.620 to 0.523)	r = 0.096 ^c^ (−0.505 to 0.635)	r = 0.265 ^c^ (−0.334 to 0.712)	r = 0.400 ^c^ (−0.262 to 0.806)

Note: Values were expressed as Pearson’s coefficient (r) and values in parentheses indicate the confidence interval. Values followed by the same letter in the same row do not differ significantly from one another (*p* > 0.05).

## Data Availability

The raw data supporting the conclusions of this article will be made available by the authors on request.

## Abbreviations

The following abbreviations are used in this manuscript:

CDV	Canine distemper virus
CNS	Central nervous system
RT-PCR	Reverse transcriptase polymerase chain reaction
NTC	No-template control
NRT	No-reverse-transcriptase control
